# Impact of Combined “CHADS-BLED” Score to Predict Short-Term Outcomes in Transfemoral and Transapical Aortic Valve Replacement

**DOI:** 10.1155/2020/9414397

**Published:** 2020-12-18

**Authors:** Verena Veulemans, Oliver Maier, Georg Bosbach, Katharina Hellhammer, Shazia Afzal, Kerstin Piayda, Amin Polzin, Christian Jung, Ralf Westenfeld, Arash Mehdiani, Artur Lichtenberg, Malte Kelm, Tobias Zeus

**Affiliations:** ^1^Division of Cardiology, Pulmonology and Vascular Medicine, Heinrich Heine University, Medical Faculty, Moorenstr. 5, Düsseldorf 40225, Germany; ^2^Division of Cardiovascular Surgery, Heinrich Heine University, Medical Faculty, Moorenstr. 5, Düsseldorf 40225, Germany; ^3^CARID (Cardiovascular Research Institute Düsseldorf), Moorenstr. 5, Düsseldorf 40225, Germany

## Abstract

**Background:**

High CHA_2_DS_2_-VASC and HAS-BLED scores are linked to increased mortality in structural and nonstructural cardiovascular interventions irrespective of the presence of atrial fibrillation (AF) or oral anticoagulation. We aimed to use the aforementioned scores to quantify the risk of 30-day mortality, major vascular and bleeding events (MVASC/BARC), and cerebrovascular insults (CVI) in patients undergoing different access routes in transcatheter aortic valve replacement (TAVR).

**Methods:**

Out of 1329 patients, 980 transfemoral (TF) TAVR (73.7%) and 349 transapical (TA) TAVR (26.3%) were included. CHA_2_DS_2_-VASC, HAS-BLED, and combined “CHADS-BLED” scores were calculated and compared to the predictive value of the established EuroSCORE and STS score.

**Results:**

In all-comers TF TAVR patients, the applied risk models showed only poor association with 30-day mortality while, in patients with concomitant AF, a strong association was observed using the combined CHADS-BLED score (c-index: 0.83; 95% CI: 0.76–0.91; *p* < 0.0001). Concerning 30-day mortality, only the STS score for TF TAVR (c-index: 0.68; 95% CI: 0.59–0.76; *p* = 0.001) and EuroSCORE for TA TAVR (c-index: 0.66; 95% CI: 0.56–0.76; *p* = 0.005) could show some predictive value. High CHADS-BLED was associated with enhanced CVI (3.0% vs. 7.2%;*p*=0.0039^*∗*^) and more frequent MVASC/BARC (3.2% vs. 6.3%; *p* = 0.0362) in the all-comers TAVR cohort. All risk models failed in the prediction of CVI and MVASC/BARC for TA TAVR patients.

**Conclusion:**

The combined CHADS-BLED score was a strong predictor for 30-day mortality in TF TAVR patients with AF. A high CHADS-BLED score showed a good predictive value for major vascular and bleeding events as well as CVI in TF TAVR patients. This study is registered at clinical trials (NCT01805739).

## 1. Introduction

Transcatheter aortic valve replacement (TAVR) is an established therapeutic option in patients with symptomatic severe aortic stenosis (AS). While the STS score and the EuroSCORE are two appropriate risk score models to ascertain patients' individual short-term mortality and morbidity after cardiac surgery [1, 2], there are no comparable tools particularly established for patients undergoing TAVR, especially in terms of different access routes. In this context, the transapical (TA) approach was shown to be associated with higher morbidity and mortality compared to transfemoral (TF) access [3, 4].

The CHA_2_DS_2_-VASC score has been demonstrated to predict the risk of cerebrovascular events in patients with [5] and without atrial fibrillation (AF) [6,7]. Additionally, the HAS-BLED score can predict the risk of major bleeding and mortality in patients using oral anticoagulation [8], even in the absence of AF [9]. Although these risk scores have not been developed for the prediction of the outcome after TAVR, many components included cover the typical TAVR patient profile. Thus, both tools are considered to be associated with enhanced mortality and morbidity in several structural [10–13] and nonstructural cardiovascular interventions [6, 7, 9]. Therefore, we sought to (i) quantify the risk of 30-day mortality, major vascular and bleeding events (MVASC/BARC), and the incidence of cerebrovascular insults (CVI) in dependence from these well-established scores, (ii) to assess their combined usage (CHADS-BLED) as short-term risk stratification tool in patients undergoing TAVR with focus on differences in access routes, namely, TF and TA approach, and (iii) to compare these results with the predictive value of traditional risk scores (EuroSCORE and STS).

## 2. Methods

### 2.1. Study Population

From 2009 to 2019, out of 1329 patients with either TF (*n* = 980, 73.7%) or TA (*n* = 349, 26.3%) TAVR, CHA_2_DS_2_-VASC and HAS-BLED scores were calculated as well as the logistic EuroSCORE I and STS-PROM scores. The combined CHADS-BLED score was calculated by adding the values of the single CHA_2_DS_2_-VASC and HAS-BLED scores. All procedures were performed according to the current guidelines between 2009 and 2019, respectively, and under local anesthesia for TF access and general anesthesia for TA access. TF TAVR was performed with different generations of either the self-expandable CoreValve System (Medtronic Inc., Minneapolis, MN) or the balloon-expandable SAPIEN System (Edwards Lifesciences, Irvine, CA). TA TAVR was predominantly performed by using the SAPIEN System (Edwards Lifesciences, Irvine, CA) or in very few cases the Engager System (Medtronic Inc., Minneapolis, MN).

All patients provided written informed consent for TAVR and the use of clinical, procedural, and follow-up data for research. The study procedures were in accordance with the Declaration of Helsinki. The Institutional Ethics Committee of the Heinrich-Heine University approved the study protocol (4080). The study is registered at clinical trials (NCT01805739).

### 2.2. Clinical Outcomes, Definitions, and Assessment

All clinical outcomes were systematically assessed using the VARC-2 consensus statement [14] and are reported accordingly. The primary study endpoints were defined as 30-day all-cause mortality, MVASC/BARC (defined as requiring a vascular surgical input, procedure-related life-threatening, disabling, or major bleeding with need for blood transfusion), and CVI (defined as an acute episode of a focal or global neurological deficit caused by ischemic, hemorrhagic, or undetermined etiology and confirmed by neurological specialist or neuroimaging). Secondary clinical endpoints were need for cardiopulmonary resuscitation, conversion to surgery, sepsis, acute kidney injury, and new permanent pacemaker insertion within 30 days of TAVI.

### 2.3. Statistical Analysis

The collected data included patients' characteristics, imaging findings, periprocedural in-hospital data, laboratory results, and follow-up data. Continuous data were described by mean and standard deviation, median, or upper and lower 95% confidence interval (interquartile ranges). Categorical variables were characterized by frequencies and percentages. Continuous variables were compared using Student's *t*-test or Kolmogorov–Smirnov test depending on variable distribution. Categorical variables were compared using Fisher's exact test. Survival was analyzed using the Kaplan–Meier plots and logrank tests. Receiver operating characteristic (ROC) analysis and the c-index (area under the curve (AUC)) were used to identify the sensitivity and specificity of the CHA_2_DS_2_-VASC, HAS-BLED, and combined CHADS-BLED cutoff points for 30-day mortality, MVASC/BARC, and CVI. The optimal cutoff values were defined by Youden's index, the point at which the value of “sensitivity + specificity − 1” was maximum.

The data analysis was performed using the statistical software SPSS (version 23.0, SPSS Inc., Chicago, IL, USA) and GraphPad Prism (version 6.0, GraphPad Software, San Diego, CA, USA). All statistical tests were 2-tailed, and a value of *p* < 0.05 was considered statistically significant.

## 3. Results

### 3.1. Baseline Characteristics: Clinical and Functional Data

Baseline patients' characteristics differed in consequence of the particular risk profile and selection bias of TF versus TA assigned approaches: TA patients were younger (TF vs. TA AVR: age 81.7 ± 5.7 vs. 78.5 ± 6.8;*p* < 0.0001) and predominantly male (TF vs. TA TAVR: female 54.5% vs. 39.3%;*p* < 0.0001). In summary, general atherosclerosis in the meaning of concomitant coronary artery disease (CAD), peripheral artery disease (PAD), cerebrovascular disease (CVD), and porcelain aorta (PAo) were more frequent in patients undergoing TA TAVR. The logistic EuroSCORE I (logES-I) was higher in TA TAVR patients according to the predescribed risk profile (TF vs. TA TAVR: logES-I 25.4 ± 15.8 vs. 27.9 ± 16.8; *p*=0.011). No difference was observed concerning mono or dual antiplatelet therapy, usage of (new) oral anticoagulants, or triple therapy at admission. A full overview of baseline clinical and functional characteristics is displayed in Supplemental Table 1.

### 3.2. Outcome Analysis in Different TAVR Access Routes

Periprocedural death within the first 30 days was recorded in 59 cases (4.4%) in the overall cohort with a mortality distribution of 3.3% (*n* = 32) in the TF TAVR and 7.7% (*n* = 27) in the TA TAVR cohort (*p* = 0.0012%). Cardiovascular death was documented in 39 cases with higher amount in patients undergoing TA approach (TF vs. TA TAVR: 2.1% vs. 5.2%; *p* = 0.0003). Further causes of overall 30-day death were cerebrovascular accidents (*n* = 3, 0.2%), infection/sepsis (*n* = 15; 1.1%), and unknown reasons (*n* = 2; 0.2%).

Cerebrovascular events were recorded in 68 cases (5.1%) in the overall cohort with a distribution of 5.3% (*n* = 52) in the TF TAVR and 4.6% (*n* = 16) in the TA TAVR cohort. There were no significant differences in both cohorts regarding the overall distribution and subclassifications. Transient ischemic attacks counted with 19 events (1.4%), ischemic strokes with 33 events (2.5%), hemorrhagic CVI with 4 events (0.3%), and undetermined CVI with 6 events (0.5%). MVASC/BARC events recorded in 146 cases (11.0%) were significantly higher in the TA TAVR cohort (TF vs. TA TAVR: 9.5% vs. 15.2%;*p* < 0.0001). Except for conversion to surgery and need for permanent pacemaker therapy, all secondary outcomes were more unfavorable in TA TAVR patients. A complete overview of primary and secondary outcomes according to VARC-2 is displayed in Supplemental Table 2.

### 3.3. CHA_2_DS_2_-VASC, HAS-BLED, and Combined CHADS-BLED Performance for Prediction of 30-Day Mortality, CVI, and MVASC/BARC (All-Comers)

The risk model discrimination performance is reported in Table 1 and Supplemental Figure 1. The aforementioned risk models showed only poor association with 30-day mortality in TF TAVR (CHA_2_DS_2_-VASC: c-index: 0.57, 95% CI: 0.47–0.68, *p* = 0.162; HAS-BLED: c-index: 0.58, 95% CI: 0.46–0.69, *p* = 0.132; CHADS-BLED: c-index: 0.60, 95% CI: 0.49–0.71, *p* = 0.058) and TA TAVR patients (CHA_2_DS_2_-VASC: c-index: 0.53, 95% CI: 0.43–0.64, *p* = 0.559; HAS-BLED: c-index: 0.58, 95% CI: 0.47–0.70, *p* = 0.157; CHADS-BLED: c-index: 0.57, 95% CI: 0.46–0.68, *p* = 0.217). Concerning 30-day mortality in all-comers TF and TA TAVR patients, only the STS score for TF TAVR (c-index: 0.675; 95% CI: 0.59–0.76; *p* = 0.001) and the EuroSCORE for TA TAVR (c-index: 0.66; 95% CI: 0.56–0.76; *p* = 0.005) could show a predictive value.

HAS-BLED (c-index: 0.66; 95% CI: 0.58–0.74;*p* < 0.0001) and CHADS-BLED (c-index: 0.66; 95% CI: 0.58–0.73;*p* < 0.0001) performed best concerning prediction of CVI.

Regarding the prediction of MVASC/BARC, HAS-BLED (c-index: 0.59; 95% CI: 0.51–0.68; *p* = 0.035) and CHADS-BLED (c-index: 0.59; 95% CI: 0.50–0.67; *p* = 0.048) were superior to CHA_2_DS_2_-VASC (c-index: 0.54; 95% CI: 0.47–0.62; *p* = 0.319) in TF TAVR patients, while the best prediction of MVASC/BARC for TF TAVR was reached by the STS score (c-index: 0.65; 95% CI: 0.58–0.73; *p* < 0.0001). Indeed, all risk models failed in the prediction of the primary endpoints in patients undergoing TA access.

Receiver operating characteristic (ROC) analysis and the c-index (area under the curve, AUC) were used to identify the sensitivity and specificity of the CHADS-BLED cutoff points for 30-day mortality, CVI, and MVASC/BARC. The optimal cutoff values were defined by Youden's index. >7 points turned out to be the cutoff with “sensitivity + specificity − 1” becoming the maximum regarding the combined CHADS-BLED calculation (see Supplemental Figure 1) in every event (30-day mortality, CVI, and MVASC/BARC) and access class (TF vs. TA TAVR).

**Figure 1:**
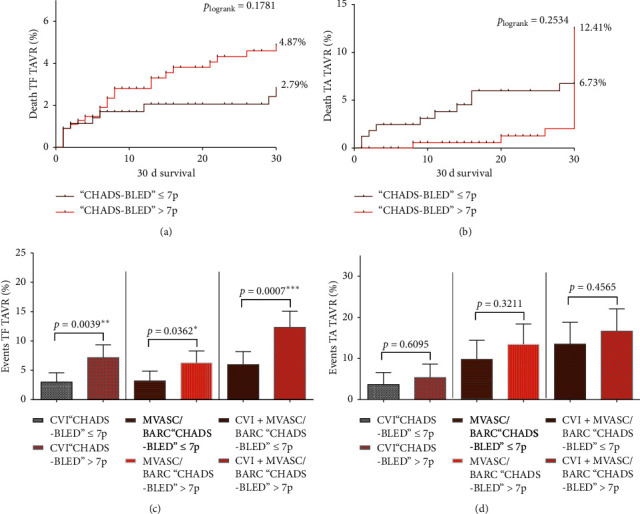
Kaplan–Meier survival curves for 30-day mortality and adverse event rates according to high and low combined CHADS-BLED score. (a) Kaplan–Meier survival curves according to the combined CHADS-BLED score in TF TAVR and (b) TA TAVR patients. (c) Awarding of adverse event rates according to high and low combined CHADS-BLED score in TF TAVR and (d) TA TAVR patients.

In the following, the Kaplan–Meier curves were plotted to clarify the impact on 30-day mortality of combined CHADS-BLED considering ≤7 points and >7 points: according to the previously established discrimination model, mortality increased in TF TAVR patients classified with more than 7 points, but was not significantly different as compared to low CHADS-BLED ≤7 points (≤7 points vs. >7 points: 2.8% vs. 4.9%; *p*_logrank_ = 0.1781, Figure 1(a)). Surprisingly, TA TAVR patients showed inverse relationship (≤7 points vs. >7 points: 12.4% vs. 6.7%; *p*_logrank_ = 0.2534, Figure 1(b)). Looking at the several event rates, high CHADS-BLED was associated with enhanced CVI (≤7 points vs. >7 points: 3.0 [1.4–4.6] vs. 7.2 [5.0–9.3]; *p*_logrank_ = 0.0039, Figure 1(c)), increased MVASC/BARC (≤7 points vs. >7 points: 3.2 [1.6–4.9] vs. 6.3 [4.2–8.3]; *p*_logrank_ = 0.0362, Figure 1(c)), and more combined events (≤7 points vs. >7 points: 6.0 [3.7–8.2] vs. 12.3 [9.5–15.1]; *p*_logrank_ = 0.0007, Figure 1(c)) in TF TAVR patients. Again, no association could be found in low vs high CHADS-BLED scoring and the incidence of the primary endpoint events in TA TAVR patients (Figure 1(d)).

### 3.4. Subanalysis of CVI and/or MVASC/BARC Positive Patients regarding Access Sites

To clarify which factors may have an impact on the adverse vascular and bleeding events in TF vs TA TAVR cohorts, CVI and/or MVASC/BARC positive patients were further analyzed towards differences in baseline characteristics, risk models, and the underlying antithrombotic regime.

As mentioned before, TA patients were predominantly male (TF vs. TA TAVR: female 64.5% vs. 39.6%; *p* < 0.0001) and less obese (TF vs. TA TAVR: BMI 27.0 ± 4.7 vs. 25.4 ± 4.3; *p* = 0.043). Concomitant CAD, PAD, and PAo were also more frequent in patients undergoing TA TAVR. While all other risk models were comparable in both groups, only the HAS-BLED was higher in TF TAVR patients (TF vs. TA TAVR: 3.5 ± 0.9 vs. 3.0 ± 1.1; *p* = 0.005). No difference was documented concerning mono or dual antiplatelet therapy, usage of (new) oral anticoagulants, or triple therapy following TAVR. A full overview of differing characteristics between TF and TA TAVR patients with CVI and/or MVASC/BARC positive profile is displayed in Supplemental Table 3.

### 3.5. Subanalysis of Patients with AF

Risk model discrimination performance is reported in Table 2 and Supplemental Figure 2. In patients with AF, the aforementioned risk models showed good association with 30-day mortality in TF TAVR. Best prediction was performed by CHADS-BLED (c-index: 0.83; 95% CI: 0.76–0.91; *p* < 0.0001), followed by CHA_2_DS_2_-VASC (c-index: 0.78; 95% CI: 0.70–0.87;*p*=0.001) and HAS-BLED (c-index: 0.74; 95% CI: 0.59–0.89;*p*=0.004), and STS score (c-index: 0.73; 95% CI: 0.60–0.87;*p*=0.006). Again, no association at all could be found for risk models in TA TAVR patients.

HAS-BLED performed best concerning prediction of CVI (c-index: 0.66; 95% CI: 0.54–0.77; *p*=0.019), while the STS score (c-index: 0.72; 95% CI: 0.61–0.84; *p*=0.001) and EuroSCORE (c-index: 0.69; 95% CI: 0.56–0.82; *p* = 0.007) were superior to HAS-BLED (c-index: 0.64; 95% CI: 0.52–0.76; *p* = 0.043) in terms of MVASC/BARC, similar to the results for all-comers TF TAVR cohort.

Once again, all risk models failed in the prediction of the primary endpoints in patients undergoing TA access.

**Figure 2:**
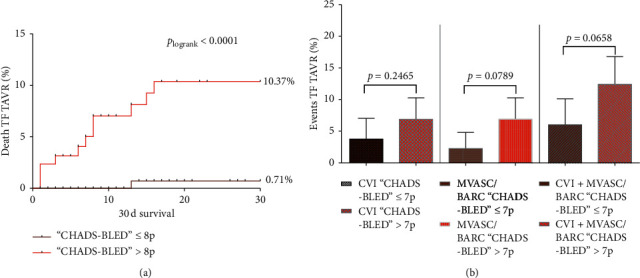
Kaplan–Meier survival curves for 30-day mortality and adverse event rates according to high and low combined CHADS-BLED score in patients with AF. (a) Kaplan–Meier survival curves according to the combined CHADS-BLED score in TF TAVR patients with AF. (b) Awarding of adverse event rates according to high and low combined CHADS-BLED score in TF TAVR patients with AF.

While the optimal cutoff values in TF patients with AF were defined by Youden's index with a CHADS-BLED >8 points for 30-day mortality and >7 points for CVI and MVASC/BARC, the Kaplan–Meier curves and cumulative statistics were plotted. Mortality was significantly different in TF patients classified with more than 8 points as compared to low CHADS-BLED ≤8 points (≤8 points vs. >8 points: 0.7% vs. 10.4%; *p*_logrank_<0.0001, Figure 2(a)). Looking at the several event rates, high CHADS-BLED was associated with increased incidence, but not significantly different from low CHADS-BLED. For further information, see also Figure 2(b). Because all scores failed in TA TAVR patients with AF, no further discrimination was established.

## 4. Discussion

The present study evaluated the risk of 30-day mortality, major vascular and bleeding events, and the incidence of CVI in dependence from the aforementioned scores in patients undergoing different access routes in TAVR and revealed several findings:

1. Only in patients with AF, the combined CHADS-BLED was superior to a single use of CHA_2_DS_2_-VASC or HAS-BLED and superior to the use of the traditional risk scores, STS and EuroSCORE, in prediction of 30-day mortality
2. In all-comers TF TAVR patients, high CHADS-BLED (>7 points) was associated with enhanced CVI and more frequent MVASC/BARC, but only to the same extent as HAS-BLED
3. The risk models failed even with higher score thresholds to predict primary endpoints in TA TAVR patients, except for EuroSCORE and STS score in terms of 30-day mortality
4. CVI and MVASC/BARC events were not linked to different antithrombotic and/or antiplatelet regimes

### 4.1. Predictive Value of CHA_2_DS_2_-VASC, HAS-BLED, and Combined CHADS-BLED

Accurate risk assessment for TAVR patients remains challenging and simple risk scores for bedside use are still lacking. Both the CHA_2_DS_2_-VASC (congestive heart failure, hypertension, age  ≥ 75 years, diabetes, prior stroke, vascular disease, age 65–74 years, and female sex) and HAS-BLED (hypertension, abnormal renal/liver function, stroke, bleeding history or predisposition, labile INR, elderly, and drugs/alcohol concomitantly) are well-established and routinely used scores for therapeutic decision support concerning patients with AF. In the past few years, these risk models were also stratified for conditions other than AF [9–13]. Hamid et al. showed a strong association between a modified R_2_CHA_2_DS_2_-VASC score and 30-day mortality. Patients with a baseline CHA_2_DS_2_-VASC score ≥6 or a modified score ≥7 appeared to have increased short-term mortality [10]. In comparison with our work, the population of Hamid et al. showed lower risk profile (log EuroSCORE 21.8 vs. 26.7) but higher 30-day mortality (7.7% vs. 4.4%), probably due to the older character of the study with first-generation devices and less experienced heart teams. Orvin et al. recently demonstrated in a three-category model that both stroke and mortality at 1 year were significantly more frequent with increasing CHA_2_DS_2_-VASC score [12]. Honda et al. showed that the HAS-BLED score could predict the risk of severe bleeding and mortality in patients who underwent TF TAVR independent of the presence of AF [13].

However, bedside score-derived prediction of mortality and adverse events in the context of different access routes comparing TF and TA TAVR has not been investigated so far. Our study is in line with former reports that TA patients show higher mortality and periprocedural adverse events than TF patients [3, 4]. Surprisingly, in our study, neither CHA_2_DS_2_-VASC nor HAS-BLED score was associated with 30-day mortality in all-comers TF and TA TAVR patients, and all risk models failed in the prediction of the primary endpoints in patients undergoing TA access. Hence, only in patients with AF, the CHADS-BLED was strongly associated with 30-day mortality. This is in close relation to the original use of the scores, namely, prediction of outcome only in patients with AF, refusing former results about their usefulness in risk prediction regardless of the presence of AF [10, 12, 13].

Looking at the several event rates, high CHADS-BLED >7 points was associated with enhanced CVI, MVASC/BARC, and combined events in TF TAVR patients, but only in the all-comers cohort (with and without AF). No association could be found in low vs high CHADS-BLED scoring and incidence of the primary endpoint events in TA TAVR patients. Former studies could show that single use of CHA_2_DS_2_-VASC is able to provide strong correlations for in-hospital stroke but with low accuracy [11], comparable to our results. Interestingly, the HAS-BLED discriminated best for CVI and not for internally predetermined bleeding events. Furthermore, despite the fact that CVI was equally distributed between TF and TA patients and that MVASC/BARC events were significantly more frequent in TA TAVR patients, this discrimination applied only in TF TAVR patients, supposing more influencing variables like concomitant antiplatelet [15, 16] or antithrombotic regimes [17]. However, subanalysis of CVI + MVASC/BARC events refused any dependency from underlying antithrombotic or antiplatelet regimes, including mono and dual antiplatelet therapy, usage of single oral anticoagulants, or combination with antiplatelet therapy.

### 4.2. Comparison to the Established STS Score and EuroSCORE

The more traditional risk scoring systems STS score and logistic EuroSCORE I have been developed and validated for the prediction of 30-day mortality and major comorbidity rates in surgical populations. They are commonly used to assess risk in patients considered for TAVR as well due to lack of proper alternatives. As expected, the STS score and EuroSCORE turned out to be most predictive for 30-day mortality in all-comers TF and TA TAVR in the present study.

Surprisingly, the STS score was superior to HAS-BLED in the prediction of MVASC/BARC events although the STS score does not contain any bleeding-specific features as HAS-BLED does. As reported in former studies, there is an effect of post-TAVI bleeding on short-term mortality. Wang et al. showed by meta-analysis that there is an about 3-fold increase in 30-day mortality associated with bleeding events, which could explain the good predictive value of the STS score in this field [18].

However, while these operative risk scores are derived from surgical aortic valve replacement, they tend to overestimate TAVR mortality because of many procedural confounders (for example, general anesthesia is not needed for TF TAVR). Furthermore, many important noncardiac factors such as frailty, malignancy, and neurological status are not part of these risk models. This is in line with our result that one-third of the deaths in our study were due to infection or sepsis, supposing various influencing noncardiac factors not being reflected by the parameters included in the current risk scores. In addition, none of the risk scores take into account anatomical factors such as vessel calcification and procedural aspects, both variables that strongly impact short-term outcome in TAVR.

These findings highlight the need of more precise scoring systems regarding the complex clinical situation of patients with severe aortic valve stenosis. This is why the current guidelines acknowledge the deficient character of the risk scores and recommend a multidisciplinary heart team-based decision for TAVR after detailed clinical evaluation with participation of patients and their families [19].

## 5. Limitations

The current study is a hypothesis-generating retrospective single-center analysis designed to test the association between single and combined use of risk scores regarding periprocedural adverse events and 30-day mortality. Due to the long time period between 2009 and 2019, there is a high variability in devices and generation of devices. With advances in technique, device, and expansion of TAVR to lower risks groups, the mortality and CVI rates have improved over time. In the present analysis, the measured outcomes were not linked to different antithrombotic and/or antiplatelet regimes. The authors cannot exclude that this absent effect is due to patients' noncompliance in medication intake although all patients had comprehensible medication plans before and after TAVR.

Further studies are warranted to validate our findings, taking other procedural factors and different risk profiles—high, intermediate, and low risk—into account. A propensity-matched score analysis should be considered to clarify the observed differences in risk model-derived prediction of adverse events in TA and TF patients.

## 6. Conclusion

The combined CHADS-BLED score was a strong predictor for 30-day mortality in TF TAVR patients with AF but failed to predict any adverse event in TA TAVR. Neither a single unified scale or scoring system nor a combination of established risk scores seems to be able to adequately predict both short-term mortality and occurrence of adverse events like MVASC/BARC or CVI in patients with aortic valve stenosis. The complex clinical situation of these patients and different access routes for TAVR require more accurate risk assessment tools, especially regarding noncardiac factors like functional status and frailty as well as anatomical and procedural factors.

## Figures and Tables

**Table 1:** Discrimination performance (ROC and AUC statistics): all-comers.

Groups/endpoints	Variables	AUC	*p* value	Lower 95% CI	Upper 95% CI
*(A) TF TAVR*					
30-day mortality	Log. EuroSCORE I	0.53	0.535	0.42	0.64
	STS score	0.68	**0.001**	0.59	0.76
	CHA_2_DS_2_-VASC	0.57	0.162	0.47	0.68
	HAS-BLED	0.58	0.132	0.46	0.69
	“CHADS-BLED”	0.60	0.058	0.49	0.71
CVI	Log. EuroSCORE I	0.53	0.488	0.49	0.61
	STS score	0.59	**0.022**	0.52	0.67
	CHA_2_DS_2_-VASC	0.60	**0.015**	0.53	0.67
	HAS-BLED	0.66	**<0.0001**	0.58	0.74
	“CHADS-BLED”	0.66	**<0.0001**	0.58	0.73
MVASC/BARC	Log. EuroSCORE I	0.55	0.246	0.46	0.64
	STS score	0.65	**<0.0001**	0.58	0.73
	CHA_2_DS_2_-VASC	0.54	0.319	0.47	0.62
	HAS-BLED	0.59	**0.035**	0.51	0.68
	“CHADS-BLED”	0.59	**0.048**	0.50	0.67

*(B) TA TAVR*					
30-day mortality	Log. EuroSCORE I	0.66	**0.005**	0.56	0.77
	STS score	0.64	**0.016**	0.55	0.73
	CHA_2_DS_2_-VASC	0.53	0.559	0.43	0.64
	HAS-BLED	0.58	0.157	0.47	0.70
	“CHADS-BLED”	0.57	0.217	0.46	0.68
CVI	Log. EuroSCORE I	0.41	0.240	0.29	0.54
	STS score	0.41	0.244	0.28	0.55
	CHA_2_DS_2_-VASC	0.64	0.054	0.52	0.76
	HAS-BLED	0.53	0.725	0.36	0.69
	“CHADS-BLED”	0.60	0.174	0.46	0.74
MVASC/BARC	Log. EuroSCORE I	0.53	0.598	0.44	0.61
	STS score	0.56	0186	0.48	0.65
	CHA_2_DS_2_-VASC	0.53	0.490	0.44	0.63
	HAS-BLED	0.49	0.866	0.39	0.59
	“CHADS-BLED”	0.54	0.462	0.44	0.63

**Table 2:** Discrimination performance (ROC and AUC statistics): subanalysis of patients with AF.

Groups/endpoints	Variables	AUC	*p* value	Lower 95% CI	Upper 95% CI
*(C) TF TAVR*					
30-day mortality	Log. EuroSCORE I	0.64	0.105	0.49	0.79
	STS score	0.73	**0.006**	0.60	0.87
	CHA_2_DS_2_-VASC	0.78	**0.001**	0.70	0.87
	HAS-BLED	0.74	**0.004**	0.59	0.89
	“CHADS-BLED”	0.83	**<0.0001**	0.76	0.91
CVI	Log. EuroSCORE I	0.52	0.720	0.40	0.65
	STS score	0.61	0.102	0.49	0.73
	CHA_2_DS_2_-VASC	0.59	0.195	0.46	0.71
	HAS-BLED	0.66	**0.019**	0.54	0.77
	“CHADS-BLED”	0.64	**0.040**	0.52	0.76
MVASC/BARC	Log. EuroSCORE I	0.69	**0.007**	0.56	0.82
	STS score	0.72	**0.001**	0.61	0.84
	CHA_2_DS_2_-VASC	0.54	0.543	0.43	0.65
	HAS-BLED	0.64	**0.043**	0.52	0.76
	“CHADS-BLED”	0.62	0.101	0.50	0.73

*(D) TA TAVR*					
30-day mortality	Log. EuroSCORE I	0.31	0.072	0.12	0.50
	STS score	0.43	0.497	0.27	0.59
	CHA_2_DS_2_-VASC	0.31	0.070	0.17	0.45
	HAS-BLED	0.40	0.356	0.22	0.58
	“CHADS-BLED”	0.31	0.077	0.17	0.45
CVI	Log. EuroSCORE I	0.45	0.82	0.26	0.64
	STS score	0.64	0.489	0.43	0.86
	CHA_2_DS_2_-VASC	0.39	0.591	0.00	0.80
	HAS-BLED	0.25	0.235	0.00	0.60
	“CHADS-BLED”	0.31	0.369	0.00	0.72
MVASC/BARC	Log. EuroSCORE I	0.42	0.336	0.30	0.54
	STS score	0.47	0.756	0.33	0.62
	CHA_2_DS_2_-VASC	0.42	0.371	0.27	0.58
	HAS-BLED	0.56	0.485	0.39	0.73
	“CHADS-BLED”	0.49	0.865	0.31	0.66

## Data Availability

The statistical data used to support the findings of this study are available from the corresponding author upon request.

## Abbreviations

AF: Atrial fibrillation
AS: Aortic stenosis
CAD: Coronary artery disease
CHADS-BLED: Combined consideration of CHA_2_DS_2_-VASC and HAS-BLED
CVD: Cerebrovascular disease
CVI: Cerebrovascular insults
EuroSCORE: European System for Cardiac Operative Risk Evaluation
LogES-I: Logistic EuroSCORE I
MVASC/BARC: Major vascular and bleeding events according to VARC-2 criteria
PAo: Porcelain aorta
PAD: Peripheral artery disease
STS (PROM): Society of Thoracic Surgeons (Predicted Risk of Mortality)
TAVR: Transcatheter aortic valve replacement
TA: Transapical access
TF: Transfemoral access
VARC-2: Valve Academic Research Consortium (2^nd^ update).

## Supplementary Materials

Supplementary Materials Supplemental Table 1: baseline clinical and functional characteristics. Supplemental Table 2: 30-day outcomes according to VARC-2. Supplemental Table 3: subanalysis of CVI and/or MVASC/BARC positive patients. Supplemental Figure 1: risk model discrimination performance for 30-day mortality, CVI, and MVASC/BARC. Comparative model discrimination (ROC curves) for patients with TF TAVR and TA TAVR only. Receiver operating characteristic (ROC) analysis and the c-index (area under the curve, AUC) were used to identify the sensitivity and specificity of the logistic EuroSCORE I, STS score, CHA_2_DS_2_-VASC, HAS-BLED, and combined “CHADS-BLED” cutoff points for 30-day mortality, CVI, and MVASC/BARC. The optimal cutoff values were defined by Youden's index, the point at which the value of “sensitivity + specificity − 1” was maximal, leading to a cutoff of >7 points regarding the combined “CHADS-BLED” calculation in every event (30-day mortality, CVI, and MVASC/BARC) and access (TF vs TA TAVR) class. Supplemental Figure 2: risk model discrimination performance for 30-day mortality, CVI, and MVASC/BARC in AF patients. Comparative model discrimination (ROC curves) for patients with AF undergoing TF TAVR and TA TAVR. Receiver operating characteristic (ROC) analysis and the c-index (area under the curve, AUC) were used to identify the sensitivity and specificity of the logistic EuroSCORE I, STS score, CHA_2_DS_2_-VASC, HAS-BLED, and combined “CHADS-BLED” cutoff points for 30-day mortality, CVI, and MVASC/BARC. The optimal cutoff values were defined by Youden's index, the point at which the value of “sensitivity + specificity − 1” was maximal, leading to a cutoff of >8 points regarding the combined “CHADS-BLED” calculation concerning 30-day mortality and >7 points for every other event (CVI and MVASC/BARC) in TF TAVR patients.
