# Supplementary material for: Impact of Combined “CHADS-BLED” Score to Predict Short-Term Outcomes in Transfemoral and Transapical Aortic Valve Replacement
Source: J Interv Cardiol. 2020 Dec 18;2020:9414397. doi: 10.1155/2020/9414397 (PMC7762668; doi:10.1155/2020/9414397)
Supplement: Supplementary Materials — Supplemental Table 1: baseline clinical and functional characteristics. Supplemental Table 2: 30-day outcomes according to VARC-2. Supplemental Table 3: subanalysis of CVI and/or MVASC/BARC positive patients. Supplemental Figure 1: risk model discrimination performance for 30-day mortality, CVI, and MVASC/BARC. Comparative model discrimination (ROC curves) for patients with TF TAVR and TA TAVR only. Receiver operating characteristic (ROC) analysis and the c-index (area under the curve, AUC) were used to identify the sensitivity and specificity of the logistic EuroSCORE I, STS score, CHA2DS2-VASC, HAS-BLED, and combined “CHADS-BLED” cutoff points for 30-day mortality, CVI, and MVASC/BARC. The optimal cutoff values were defined by Youden's index, the point at which the value of “sensitivity + specificity − 1” was maximal, leading to a cutoff of >7 points regarding the combined “CHADS-BLED” calculation in every event (30-day mortality, CVI, and MVASC/BARC) and access (TF vs TA TAVR) class. Supplemental Figure 2: risk model discrimination performance for 30-day mortality, CVI, and MVASC/BARC in AF patients. Comparative model discrimination (ROC curves) for patients with AF undergoing TF TAVR and TA TAVR. Receiver operating characteristic (ROC) analysis and the c-index (area under the curve, AUC) were used to identify the sensitivity and specificity of the logistic EuroSCORE I, STS score, CHA2DS2-VASC, HAS-BLED, and combined “CHADS-BLED” cutoff points for 30-day mortality, CVI, and MVASC/BARC. The optimal cutoff values were defined by Youden's index, the point at which the value of “sensitivity + specificity − 1” was maximal, leading to a cutoff of >8 points regarding the combined “CHADS-BLED” calculation concerning 30-day mortality and >7 points for every other event (CVI and MVASC/BARC) in TF TAVR patients. [file 9414397.f1.zip › 9414397.f1/SupplementalFigure1.pptx]

## Slide 1
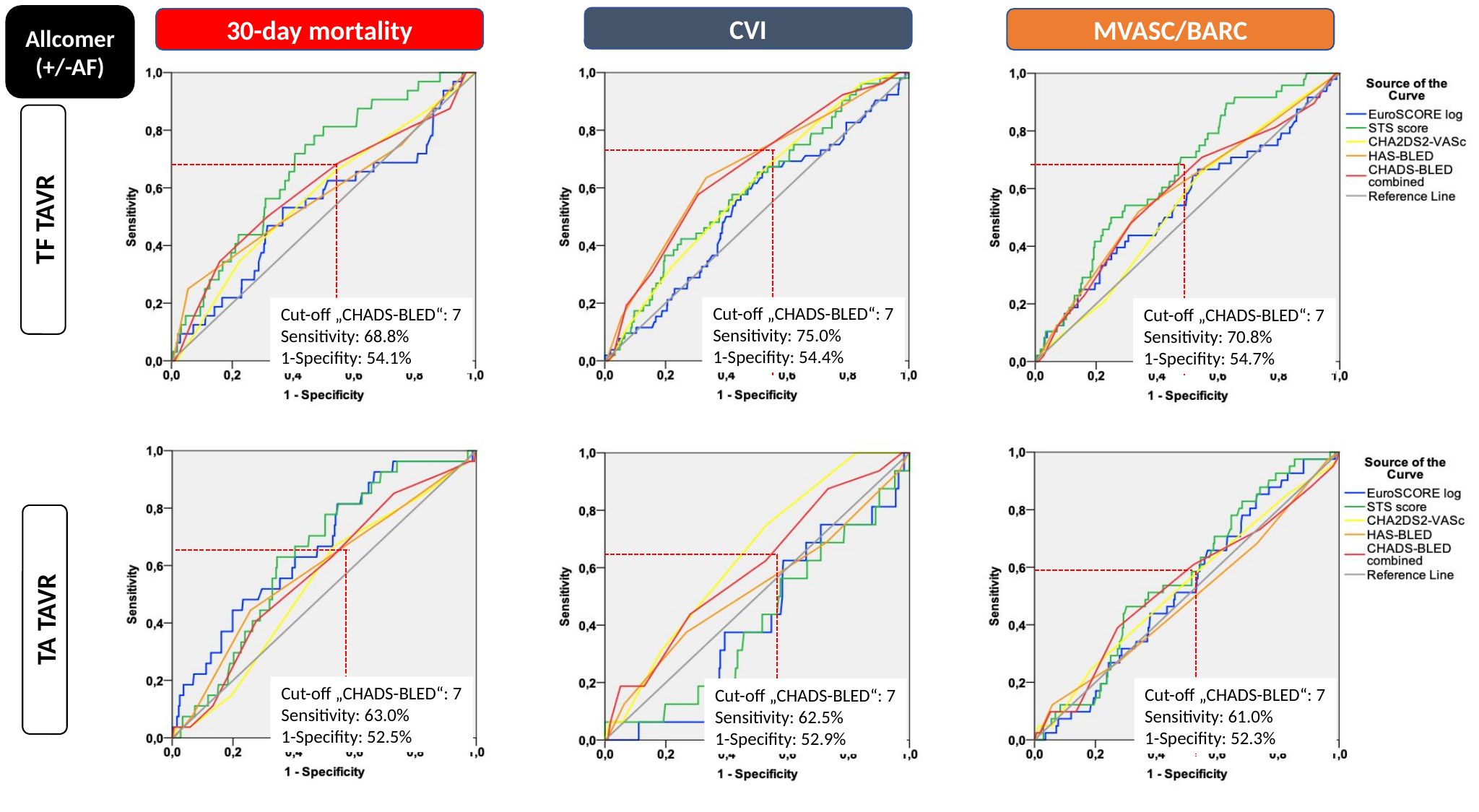

Allcomer (+/-AF)
CVI
30-day mortality
MVASC/BARC
TF TAVR
Cut-off „CHADS-BLED“: 7
Sensitivity: 75.0%
1-Specifity: 54.4%
Cut-off „CHADS-BLED“: 7
Sensitivity: 68.8%
1-Specifity: 54.1%
Cut-off „CHADS-BLED“: 7
Sensitivity: 70.8%
1-Specifity: 54.7%
TA TAVR
Cut-off „CHADS-BLED“: 7
Sensitivity: 63.0%
1-Specifity: 52.5%
Cut-off „CHADS-BLED“: 7
Sensitivity: 61.0%
1-Specifity: 52.3%
Cut-off „CHADS-BLED“: 7
Sensitivity: 62.5%
1-Specifity: 52.9%
