# Supplementary material for: Impact of Combined “CHADS-BLED” Score to Predict Short-Term Outcomes in Transfemoral and Transapical Aortic Valve Replacement
Source: J Interv Cardiol. 2020 Dec 18;2020:9414397. doi: 10.1155/2020/9414397 (PMC7762668; doi:10.1155/2020/9414397)
Supplement: Supplementary Materials — Supplemental Table 1: baseline clinical and functional characteristics. Supplemental Table 2: 30-day outcomes according to VARC-2. Supplemental Table 3: subanalysis of CVI and/or MVASC/BARC positive patients. Supplemental Figure 1: risk model discrimination performance for 30-day mortality, CVI, and MVASC/BARC. Comparative model discrimination (ROC curves) for patients with TF TAVR and TA TAVR only. Receiver operating characteristic (ROC) analysis and the c-index (area under the curve, AUC) were used to identify the sensitivity and specificity of the logistic EuroSCORE I, STS score, CHA2DS2-VASC, HAS-BLED, and combined “CHADS-BLED” cutoff points for 30-day mortality, CVI, and MVASC/BARC. The optimal cutoff values were defined by Youden's index, the point at which the value of “sensitivity + specificity − 1” was maximal, leading to a cutoff of >7 points regarding the combined “CHADS-BLED” calculation in every event (30-day mortality, CVI, and MVASC/BARC) and access (TF vs TA TAVR) class. Supplemental Figure 2: risk model discrimination performance for 30-day mortality, CVI, and MVASC/BARC in AF patients. Comparative model discrimination (ROC curves) for patients with AF undergoing TF TAVR and TA TAVR. Receiver operating characteristic (ROC) analysis and the c-index (area under the curve, AUC) were used to identify the sensitivity and specificity of the logistic EuroSCORE I, STS score, CHA2DS2-VASC, HAS-BLED, and combined “CHADS-BLED” cutoff points for 30-day mortality, CVI, and MVASC/BARC. The optimal cutoff values were defined by Youden's index, the point at which the value of “sensitivity + specificity − 1” was maximal, leading to a cutoff of >8 points regarding the combined “CHADS-BLED” calculation concerning 30-day mortality and >7 points for every other event (CVI and MVASC/BARC) in TF TAVR patients. [file 9414397.f1.zip › 9414397.f1/Supplemental-File_Table1.docx]

**Supplemental Table 1.** Baseline clinical and functional characteristics**.**

| **Clinical data** | **Over-all**  **(n=1329)** | **TF TAVR (n=980)** | **TA TAVR (n=349)** | **p-value** |
| --- | --- | --- | --- | --- |
| Age, years | 80.1 ± 6.3 | 81.7 ± 5.7 | 78.5 ± 6.8 | ***<0.0001*** |
| Female | 671 (50.5) | 534 (54.5) | 137 (39.3) | ***<0.0001*** |
| BMI | 26.5 ± 4.8 | 26.8 ± 5.2 | 26.2 ± 4.4 | ***0.043*** |
| CAD | 967 (72.8) | 682 (69.6) | 285 (81.7) | ***<0.0001*** |
| Previous PCI | 537 (40.4) | 382 (39.0) | 155 (44.4) | 0.070 |
| Previous CABG | 227 (17.1) | 128 (13.1) | 99 (28.4) | ***<0.0001*** |
| Previous valve | 33 (2.5) | 23 (2.3) | 10 (2.9) | 0.592 |
| Porcelain aorta | 176 (13.2) | 78 (8.0) | 98 (28.1) | ***<0.0001*** |
| Diabetes mellitus | 417 (31.4) | 300 (30.6) | 117 (33.5) | 0.341 |
| AHT | 1241 (93.4) | 907 (92.6) | 334 (95.7) | ***0.042*** |
| PHT | 840 (63.2) | 640 (65.3) | 200 (57.3) | ***0.011*** |
| PAD | 483 (36.3) | 245 (25.0) | 238 (68.2) | ***<0.0001*** |
| CVD | 263 (19.8) | 153 (15.6) | 110 (31.5) | ***<0.0001*** |
| Previous RRT | 66 (5.0) | 39 (4.0) | 27 (7.7) | ***0.006*** |
| COPD | 415 (31.2) | 289 (29.5) | 126 (36.1) | ***0.022*** |
| Atrial fibrillation | 470 (35.4) | 351 (35.8) | 119 (34.1) | 0.564 |
| Previous PM | 201 (15.1) | 153 (15.6) | 48 (13.8) | 0.406 |
| DPT | 220 (16.6) | 163 (16.6) | 57 (16.3) | 0.897 |
| Triple | 59 (4.4) | 44 (4.5) | 15 (4.3) | 0.881 |
| **Functional data** |  |  |  |  |
| AVA, cm^2^ | 0.7 ± 0.2 | 0.7 ± 0.2 | 0.7 ± 0.2 | 0.061 |
| dPmax (mmHg) | 60.2 ± 25.2 | 60.9 ± 25.9 | 59.5 ± 24.4 | 0.441 |
| dPmean (mmHg) | 36.3 ± 16.3 | 37.1 ± 16.3 | 35.4 ± 16.3 | 0.147 |
| CI, l/min/m^2^ | 2.3 ± 0.6 | 2.3 ± 0.6 | 2.3 ± 0.6 | 0.455 |
| **Risk models** |  |  |  |  |
| logES-I (%) | 26.7 ± 16.3 | 25.4 ± 15.8 | 27.9 ± 16.8 | ***0.011*** |
| STS-PROM | 7.1 ± 6.3 | 6.7 ± 6.0 | 7.4 ± 6.6 | 0.055 |
| CHA_2_DS_2_-VASC | 4.7 ± 1.3 | 4.7 ± 1.3 | 4.6 ± 1.2 | 0.387 |
| HAS-BLED | 3.1 ± 1.0 | 3.1 ± 1.0 | 3.0 ± 1.0 | 0.080 |
| “CHADS-BLED” | 7.7 ± 1.9 | 7.8 ± 1.9 | 7.6 ± 1.8 | 0.136 |
| Values are mean ± SD, median or n (%).  AF=atrial fibrillation; AHT=arterial hypertension; AVA=aortic valve area; BMI=body mass index; CABG=coronary artery bypass graft; CAD=coronary artery disease; CI=cardiac index; COPD=chronic obstructive pulmonary disease; CVD=cerebrovascular disease; DPT=dual antiplatelet therapy; MPT=mono platelet therapy; (N)OAC=(new) oral anticoagulants; dPmean/max=mean/max. transvalvular gradient; PCI=percutaneous coronary intervention; PHT=pulmonary hypertension; PAD=peripheral artery disease; RRT=renal replacement therapy; | | | | |
