# Supplementary material for: Impact of Combined “CHADS-BLED” Score to Predict Short-Term Outcomes in Transfemoral and Transapical Aortic Valve Replacement
Source: J Interv Cardiol. 2020 Dec 18;2020:9414397. doi: 10.1155/2020/9414397 (PMC7762668; doi:10.1155/2020/9414397)
Supplement: Supplementary Materials — Supplemental Table 1: baseline clinical and functional characteristics. Supplemental Table 2: 30-day outcomes according to VARC-2. Supplemental Table 3: subanalysis of CVI and/or MVASC/BARC positive patients. Supplemental Figure 1: risk model discrimination performance for 30-day mortality, CVI, and MVASC/BARC. Comparative model discrimination (ROC curves) for patients with TF TAVR and TA TAVR only. Receiver operating characteristic (ROC) analysis and the c-index (area under the curve, AUC) were used to identify the sensitivity and specificity of the logistic EuroSCORE I, STS score, CHA2DS2-VASC, HAS-BLED, and combined “CHADS-BLED” cutoff points for 30-day mortality, CVI, and MVASC/BARC. The optimal cutoff values were defined by Youden's index, the point at which the value of “sensitivity + specificity − 1” was maximal, leading to a cutoff of >7 points regarding the combined “CHADS-BLED” calculation in every event (30-day mortality, CVI, and MVASC/BARC) and access (TF vs TA TAVR) class. Supplemental Figure 2: risk model discrimination performance for 30-day mortality, CVI, and MVASC/BARC in AF patients. Comparative model discrimination (ROC curves) for patients with AF undergoing TF TAVR and TA TAVR. Receiver operating characteristic (ROC) analysis and the c-index (area under the curve, AUC) were used to identify the sensitivity and specificity of the logistic EuroSCORE I, STS score, CHA2DS2-VASC, HAS-BLED, and combined “CHADS-BLED” cutoff points for 30-day mortality, CVI, and MVASC/BARC. The optimal cutoff values were defined by Youden's index, the point at which the value of “sensitivity + specificity − 1” was maximal, leading to a cutoff of >8 points regarding the combined “CHADS-BLED” calculation concerning 30-day mortality and >7 points for every other event (CVI and MVASC/BARC) in TF TAVR patients. [file 9414397.f1.zip › 9414397.f1/Supplemental-File_Table2.docx]

**Supplemental Table 2.** 30-day outcomes according to VARC-2.

| **Primary clinical outcome** | **Over-all** | **TF TAVR** | **TA TAVR** | **p-value** |
| --- | --- | --- | --- | --- |
| **30-day mortality** | 59 (4.4) | 32 (3.3) | 27 (7.7) | ***0.0012*** |
| Cardiovascular death | 39 (2.9) | 18 (2.1) | 21 (5.2) | ***0.0003*** |
| None-cardiovascular death | 20 (1.5) | 10 (1.0) | 10 (2.9) | ***0.0210*** |
| - cerebrovascular | 3 (0.2) | 2 (0.2) | 1 (0.2) | 1.0000 |
| - infection/sepsis | 15 (1.1) | 7 (0.7) | 8 (2.3) | ***0.0327*** |
| Unknown reasons | 2 (0.2) | 1 (0.1) | 1 (0.2) | 1.0000 |
| **CVI** | 68 (5.1) | 52 (5.2) | 16 (4.6) | 0.6727 |
| - TIA | 19 (1.4) | 15 (1.5) | 4 (1.1) | 0.7944 |
| - Ischemic | 33 (2.5) | 25 (2.6) | 8 (2.3) | 1.0000 |
| - hemorrhagic | 4 (0.3) | 3 (0.3) | 1 (0.2) | 1.0000 |
| - undetermined | 6 (0.5) | 4 (0.4) | 2 (0.6) | 0.6555 |
| **MVASC/BARC** | 89 (6.7) | 48 (4.9) | 41 (11.7) | ***<0.0001*** |
| **Secondary clinical outcomes** |  |  |  |  |
| CPR | 57 (4.3) | 29 (3.0) | 28 (8.0) | ***0.0002*** |
| Conversion to surgery | 5 (0.5) | 3 (0.3) | 2 (0.6) | 0.6112 |
| Sepsis | 44 (3.3) | 17 (1.7) | 27 (7.7) | ***<0.0001*** |
| AKI (Stage I-III) | 51 (3.8) | 19 (1.9) | 32 (9.2) | ***<0.0001*** |
| New Pacemaker | 189 (14.2) | 158 (16.6) | 31 (8.9) | ***<0.0001*** |
| Values are n (%), p-values are calculated by fishers’ exact test  CPR=cardiopulmonary resuscitation; AKI=acute kidney injury. | | | | |
