# Supplementary material for: Impact of Combined “CHADS-BLED” Score to Predict Short-Term Outcomes in Transfemoral and Transapical Aortic Valve Replacement
Source: J Interv Cardiol. 2020 Dec 18;2020:9414397. doi: 10.1155/2020/9414397 (PMC7762668; doi:10.1155/2020/9414397)
Supplement: Supplementary Materials — Supplemental Table 1: baseline clinical and functional characteristics. Supplemental Table 2: 30-day outcomes according to VARC-2. Supplemental Table 3: subanalysis of CVI and/or MVASC/BARC positive patients. Supplemental Figure 1: risk model discrimination performance for 30-day mortality, CVI, and MVASC/BARC. Comparative model discrimination (ROC curves) for patients with TF TAVR and TA TAVR only. Receiver operating characteristic (ROC) analysis and the c-index (area under the curve, AUC) were used to identify the sensitivity and specificity of the logistic EuroSCORE I, STS score, CHA2DS2-VASC, HAS-BLED, and combined “CHADS-BLED” cutoff points for 30-day mortality, CVI, and MVASC/BARC. The optimal cutoff values were defined by Youden's index, the point at which the value of “sensitivity + specificity − 1” was maximal, leading to a cutoff of >7 points regarding the combined “CHADS-BLED” calculation in every event (30-day mortality, CVI, and MVASC/BARC) and access (TF vs TA TAVR) class. Supplemental Figure 2: risk model discrimination performance for 30-day mortality, CVI, and MVASC/BARC in AF patients. Comparative model discrimination (ROC curves) for patients with AF undergoing TF TAVR and TA TAVR. Receiver operating characteristic (ROC) analysis and the c-index (area under the curve, AUC) were used to identify the sensitivity and specificity of the logistic EuroSCORE I, STS score, CHA2DS2-VASC, HAS-BLED, and combined “CHADS-BLED” cutoff points for 30-day mortality, CVI, and MVASC/BARC. The optimal cutoff values were defined by Youden's index, the point at which the value of “sensitivity + specificity − 1” was maximal, leading to a cutoff of >8 points regarding the combined “CHADS-BLED” calculation concerning 30-day mortality and >7 points for every other event (CVI and MVASC/BARC) in TF TAVR patients. [file 9414397.f1.zip › 9414397.f1/Supplemental-File_Table3.docx]

**Supplemental Table 3.** Sub-analysis of CVI and/or MVASC/BARC positive patients.

| **Clinical data** | **Over-all**  **(n=146)** | **TF TAVR (n=93)** | **TA TAVR (n=53)** | **p-value** |
| --- | --- | --- | --- | --- |
| Female | 81 (55.5) | 60 (64.5) | 21 (39.6) | ***<0.0001*** |
| BMI | 26.2 ± 4.5 | 27.0 ± 4.7 | 25.4 ± 4.3 | ***0.043*** |
| CAD | 105 (71.9) | 62 (66.7) | 43 (81.1) | ***0.037*** |
| Porcelain aorta | 29 (19.9) | 13 (14.0) | 16 (30.2) | ***0.015*** |
| PAD | 64 (43.8) | 28 (30.1) | 36 (67.9) | ***<0.0001*** |
| **Antithrombotic regime(s)** |  |  |  |  |
| MPT | 14 (9.6) | 7 (7.5) | 7 (13.2) | 0.250 |
| DPT | 58 (39.7) | 37 (39.8) | 21 (39.6) | 1.000 |
| (N)OAC mono | 17 (11.6) | 11 (11.8) | 6 (11.3) | 1.000 |
| OAC+MPT | 17 (11.6) | 10 (10.8) | 7 (13.2) | 0.599 |
| Triple | 11 (7.5) | 7 (7.5) | 4 (7.5) | 1.000 |
| Unknown | 5 (3.4) | 2 (1.4) | 3 (5.7) | 0.347 |
| **Risk models** |  |  |  |  |
| logES-I (%) | 26.7 ± 16.3 | 27.1 ± 17.3 | 26.3 ± 13.8 | 0.766 |
| STS-PROM | 8.2 ± 7.5 | 8.3 ± 6.2 | 8.0 ± 8.7 | 0.802 |
| CHA_2_DS_2_-VASC | 4.9 ± 1.2 | 4.9 ± 1.1 | 4.8 ± 1.3 | 0.548 |
| HAS-BLED | 3.3 ± 1.0 | 3.5 ± 0.9 | 3.0 ± 1.1 | ***0.005*** |
| “CHADS-BLED” | 8.1 ± 1.9 | 8.4 ± 1.7 | 7.8 ± 2.0 | 0.060 |
| Values are mean ± SD, median or n (%).  BMI=body mass index; CAD=coronary artery disease; DPT=dual antiplatelet therapy; MPT=mono platelet therapy; (N)OAC=(new) oral anticoagulants; PAD=peripheral artery disease**.** | | | | |
